# Novel monoclonal antibodies against Fiber-1 of duck adenovirus 3 and their B cell epitopes

**DOI:** 10.3389/fvets.2022.1003262

**Published:** 2022-10-12

**Authors:** Hongxia Shao, Wenyuan Zhang, Yun Lin, Jing Xie, Dan Ren, Quan Xie, Tuofan Li, Zhimin Wan, Aijian Qin, Jianqiang Ye

**Affiliations:** ^1^Key Laboratory of Jiangsu Preventive Veterinary Medicine, Key Laboratory for Avian Preventive Medicine, Ministry of Education, College of Veterinary Medicine, Yangzhou University, Yangzhou, China; ^2^Jiangsu Co-innovation Centre for Prevention and Control of Important Animal Infectious Diseases and Zoonoses, Yangzhou, China; ^3^Joint International Research Laboratory of Agriculture and Agri-Product Safety, The Ministry of Education of China, Yangzhou University, Yangzhou, China

**Keywords:** duck adenovirus 3, Fiber-1, monoclonal antibodies, IFA, Western blot, immunoprecipitation, epitope mapping

## Abstract

Recently, the outbreak of the infection of Duck adenovirus 3 (DAdV-3) characterized by swelling and hemorrhagic liver and kidney has caused huge economic losses to duck industry since 2014 in China. To date, the B cell epitopes in the Fiber-1 protein and the underlying infection mechanism of DAdV-3 have not been investigated. In this study, the recombinant Fiber-1 protein was first expressed in *E. coli* and *six* novel monoclonal antibodies (mAbs) against Fiber-1 were generated, designated as 1D8, 1E6, 3G6, 4G1, 4G2, and 6F10, respectively. Moreover, mAbs 3G6 and 6F10 could efficiently immunoprecipitate the Fiber-1 in LMH cells infected with DAdV-3 or transfected with pcDNA3.1-Fiber-1. Notably, mAbs 3G6 and 4G2 also showed certain neutralizing activity against DAdV-3 infection *in vitro*. Epitopes mapping revealed that the B cell epitope recognized by 6F10, 3G6, 4G1, 1D8, 4G2, and 1E6 was located in 34-66aa, 67-99aa, 64-296aa, 297-329aa, 330-362aa, and 363-395aa, respectively. Sequence alignments further found that the six epitopes recognized by these mAbs were highly conserved among different DAdV-3 isolates. The generated mAbs specific to Fiber-1 and their defined epitopes provide powerful tools for establishing rapid and efficient diagnostics for the detection of DAdV-3 and pave the way for further studying on the critical role of Fiber-1 in mediating the infection of DAdV-3.

## Introduction

According to the International Committee on Taxonomy of Viruses (ICTV) classification, duck-derived adenoviruses were classified into Duck atadenovirus A and Duck aviadenovirus B. Duck adenovirus 1 (DAdV-1), belonging to Duck atadenovirus A, was also known as egg drop syndrome virus (1, 2). In 1982, Bouquet reported the infection of duck adenovirus 2 (DAdV-2) in Muscovy ducks in France (3). The affected Muscovy ducks were thin and lame clinically. In 2014, Ana Marek uncovered the sequence of DAdV-2 and DAdV-2 was then assigned to the species Duck aviadenovirus B in the genus *Aviadenovirus* based on the sequence alignment and phylogenetic analysis (4). DAdV-3, as a novel number of *Aviadenovirus*, was isolated from Muscovy ducks and reported in 2014 for the first time in southern China (5). In addition, DAdV-4 (genus *Aviadenovirus*) was recently isolated from Jinding Ducks with salpingitis in China (6). Epidemiological assay revealed that DAdV-3 has widely spread and circulated in Fujian, Zhejiang, Anhui and Guangdong provinces in China since 2014 (7, 8). The ducks infected with DAdV-3 were characterized by necrosis and hemorrhage in the liver with a morbidity rate of 40–55% and a mortality rate of 35–43%, which had caused huge economic losses to the duck industry (5, 7). Notably, the clinical co-infection of DAdV-3 with other pathogens increases the difficulty of the epidemic prevention and control of the disease caused by DAdV-3 (8).

The genome of DAdV-3 encodes three major surface structural proteins including Fiber, Hexon and Penton (9). Like the serotype 4 fowl adenovirus (FAdV-4), DAdV-3 has two Fiber proteins (Fiber-1 and Fiber-2). Our previous studies reported that Fiber-1, but not Fiber-2, directly mediated the infection of FAdV-4 (10). However, little is known about the epitopes in Fiber-1 and the molecular mechanism of the infection of DAdV-3 mediated by Fiber remains unclear. In this study, six novel B cell epitopes in Fiber-1 of DAdV-3 by using mAbs were identified and the activities of these mAbs were also characterized.

## Materials and methods

### Cells, viruses, and animals

SP2/0 myeloma cells from our laboratory were cultured in HT medium at 37°C in 5% CO_2_ incubator and LMH cells from ATCC were maintained in DMEM/F12 (Gibco, NY, USA) medium. All culture mediums were supplemented with 10% fetal bovine serum (Lonsera, Shanghai, China) and antibiotics (100 ug/mL of streptomycin and 100 U/ml of penicillin). DAdV-3 was isolated and stored in our laboratory. BALB/c mice (6-week-old) were purchased from Yangzhou University Institute of Comparative Medicine.

### Expression and purification of the recombinant Fiber-1 protein

The ORF sequence of *fiber-1* gene of DAdV-3 was amplified according to the primers listed in Table 1 and cloned into pCold-I plasmid. After sequencing, the positive recombinant plasmid was transformed into an *E. coli* BL21 strain, and the expression of Fiber-1 protein was induced for 16 h at 16°C by isopropyl β-D-1-thiogalactopyranoside (IPTG) with the final concentration of 1 mM. For the purification, the recombinant Fiber-1 protein expressed in the inclusion bodies of bacterial cells was washed with buffer I (50 mM Tris-HCl, 100 mM EDTA, 100 mM NaCl, 1%Triton-X100, pH 8.0) and buffer II (50 mM Tris-HCl, 1 mM EDTA, 50 mM NaCl, 1%Triton-X100, 100 mM DTT, 2M Urea, pH 8.0). Then the precipitate turned into soluble protein using urea dialysis and was evaluated by SDS-PAGE and Western blot analysis.

**Table 1:** Primers for amplifying the linear plasmid, *fiber-1* gene and its truncations.

**Name**	**Direction**	**Sequence (5^′^–3^′^)**
pCold-1-F1-F	Forward	CCGCTCGAGATGCTCTGTCCGTTTAGATTCATCC
pCold-1-F1-R	Reverse	CCGGAATTCTTATACAATCTTCGCTAGGTA
pc-F	Forward	GAATTCTGCAGATATCCAGCACAGTG
pc-R	Reverse	GCTCGGTACCAAGCTTAAGTTTAAACG
pcDNA3.1-F1-1-99-F	Forward	AGCTTGGTACCGAGCATGCTCTGTCCGTTTAGATT
pcDNA3.1-F1-1-99-R	Reverse	ATATCTGCAGAATTCTTAGTCACTGTTGGCCCGGC
pcDNA3.1-F1-1-198-R	Reverse	ATATCTGCAGAATTCTTAAGGCGGCGGAGGTGTAG
pcDNA3.1-F1-1-297-R	Reverse	ATATCTGCAGAATTCTTAGCAGCTACCACCCCCAC
pcDNA3.1-F1-1-396-R	Reverse	ATATCTGCAGAATTCTTACACAGCTTTTGAGCCTG
pcDNA3.1-F1-1-495-R	Reverse	ATATCTGCAGAATTCTTAGTCGAGTGTGACACCAC
pcDNA3.1-F1-1-594-R	Reverse	ATATCTGCAGAATTCTTATGTGACTATGTCTAGAC
pcDNA3.1-F1-1-690-R	Reverse	ATATCTGCAGAATTCTTAATCGACTGCAACTCTTATTC
pcDNA3.1-F1-691-789-F	Forward	AGCTTGGTACCGAGCATGGGAACGACAGTAAAAGT
pcDNA3.1-F1-691-789-R	Reverse	ATATCTGCAGAATTCTTAGTCTGATTCTGGTGATC
pcDNA3.1-F1-691-888-R	Reverse	ATATCTGCAGAATTCTTAGATGACACCGTTCACCA
pcDNA3.1-F1-691-987-R	Reverse	ATATCTGCAGAATTCTTAAAATTGTTTGTCGAACG
pcDNA3.1-F1-691-1086-R	Reverse	ATATCTGCAGAATTCTTACCCTGTGAATCCACCGA
pcDNA3.1-F1-691-1185-R	Reverse	ATATCTGCAGAATTCTTAAGTCGCGGGGAAAAATT
pcDNA3.1-F1-691-1284-R	Reverse	ATATCTGCAGAATTCTTAAAACGAAAAAGCAAGAG
pcDNA3.1-F1-691-1380-R	Reverse	ATATCTGCAGAATTCTTATACAATCTTCGCTAGGT

### Preparation of polyclonal antibody against Fiber-1

Fifty microgram of the purified Fiber-1 protein mixed with Freund's complete adjuvant (Sigma-Aldrich, Missoula, MO, USA) was immunized to BALB/c mice (6-week-old) through intraperitoneal injection. After an interval of 2 weeks, the following two immunizations were administered with 50 μg Fiber-1 protein mixed with Freund's incomplete adjuvant (Sigma-Aldrich, Missoula, MO, USA) at an interval of 10 days. The mouse blood was collected to prepare polyclonal antibody at day 10 after the last immunization. The antibody titer of the serum against Fiber-1 was measured through IFA.

### Generation of mAbs against Fiber-1

The mouse with the highest antibody titer against Fiber-1 was boosted with 50 μg of the purified Fiber-1 protein. Three days after the final immunization, the spleen cells of the boosted mouse were fused with SP2/0 myeloma cells by polyethylene glycol 1500 (Roche, Mannheim, Germany). The fused cells were maintained in HAT selective medium for 10 days, and then the culture supernatants were screened for mAbs against Fiber-1 using LMH cells infected with DAdV-3 as antigen through IFA. Positive hybridoma clones secreting antibodies against Fiber-1 were subcloned three times by limiting dilution assay. The ascites fluids containing mAbs were generated using female BALB/c mice and purified using protein G column (GE Healthcare Life sciences, Uppsala, Sweden). The isotypes of mAbs were determined using a commercial mouse mAb isotyping kit (Thermo Scientific, Massachusetts, USA) according to the manufacturer's protocol.

### Indirect immunofluorescent assay

LMH cells were infected with DAdV-3 at 0.1 MOI or transfected with pcDNA3.1-Fiber-1 and its different truncations, respectively. At day 3 post-infection or day 2 post-transfection, the cells were fixed with precooled acetone and ethanol (3:2) for 5 min at room temperature. The fixed cells were incubated with mAbs of indicated dilutions for 45 min at 37°C and washed three times with PBS. Then the cells were incubated with FITC-conjugated goat anti-mouse IgG (Sigma-Aldrich, USA) at a dilution of 1:150 for 45 min at 37°C, followed by three washes with PBS. Finally, the cells were observed using an inverted fluorescence microscope.

### Western blot

LMH cells infected with DAdV-3 or transfected with pcDNA3.1-Fiber-1 were collected and lysed in lysis buffer with protease and phosphatase inhibitors (CST, MA, USA). The lysates mixed with the loading buffer were boiled and separated *via* SDS-PAGE. The gel was then transferred onto nitrocellulose membranes (NCs) (GE Healthcare Life sciences, Freiburg, Germany). After blocked with 5% skim milk for 2 h at room temperature (RT), the NCs were washed three times with PBST. The mAbs prepared in this study and mouse anti-GAPDH monoclonal antibody (Abclonal, Wuhan, China) were served as the primary antibody, and HRP-conjugated goat anti-mouse IgG was used as the secondary antibody. Following three washes with PBST, the NCs were visualized using an automatic chemiluminescence imaging system (Tanon 5200).

### Immunoprecipitation

LMH cells infected with DAdV-3 or transfected with pcDNA3.1-Fiber-1 were collected and lysed in lysis buffer with protease and phosphatase inhibitors (CST, MA, USA). The lysates were then mixed with 2 μg of the purified mAbs generated in this study on a four-dimensional rotation at 4°C overnight. The second day, the mixture was incubated with 25 μL of protein G-Sepharose beads (Beyotime) at 4°C for 3 h to form stable complex of antigen, antibody and beads. After washed five times with PBS, the complex was boiled in the loading buffer for Western blot assay. mAb 1D8 was served as the primary antibody for detecting the specific band of Fiber-1.

### Neutralization assay

To further determine the neutralizing activity of mAbs generated here, serial diluted mAbs were incubated with 10^3^ TCID_50_ of DAdV-3 for 1 h at 37°C. The virus-mAb mixture was incubated with LMH cells for 2 h in a 6-well plate and then the culture supernatant was discarded. The infected and uninfected LMH cells were maintained in DMEM/F12 medium with 1% fetal bovine serum. After 72 h post-infection, the cells were lysed and analyzed by Western blot assay as described above. The mAb 3G12 against HA of H9N2 influenza virus generated and kept in our laboratory was used the control mAb in the neutralization assay.

### Mapping of B cell epitopes recognized by mAbs

The *fiber-1* and different *fiber-1* truncations were amplified using corresponding primers listed in Table 1 and cloned into linear pcDNA3.1 using ClonExpressTM II kit (Vazyme, Nanjing, China) to generate pcDNA3.1-Fiber-1 and other *fiber-1* truncations. These recombinant plasmids were expressed in LMH cells and the epitopes recognized by the mAbs were identified using IFA. To evaluate the conservation of the identified B cell epitopes in Fiber-1, the sequences of Fiber-1 among different DAdV-3 and other representive DAdV isolates deposited in Genbank were aligned using DNASTAR Megalign software.

## Results

### Expression and purification of the recombinant Fiber-1 of DAdV-3

**Figure 1:**
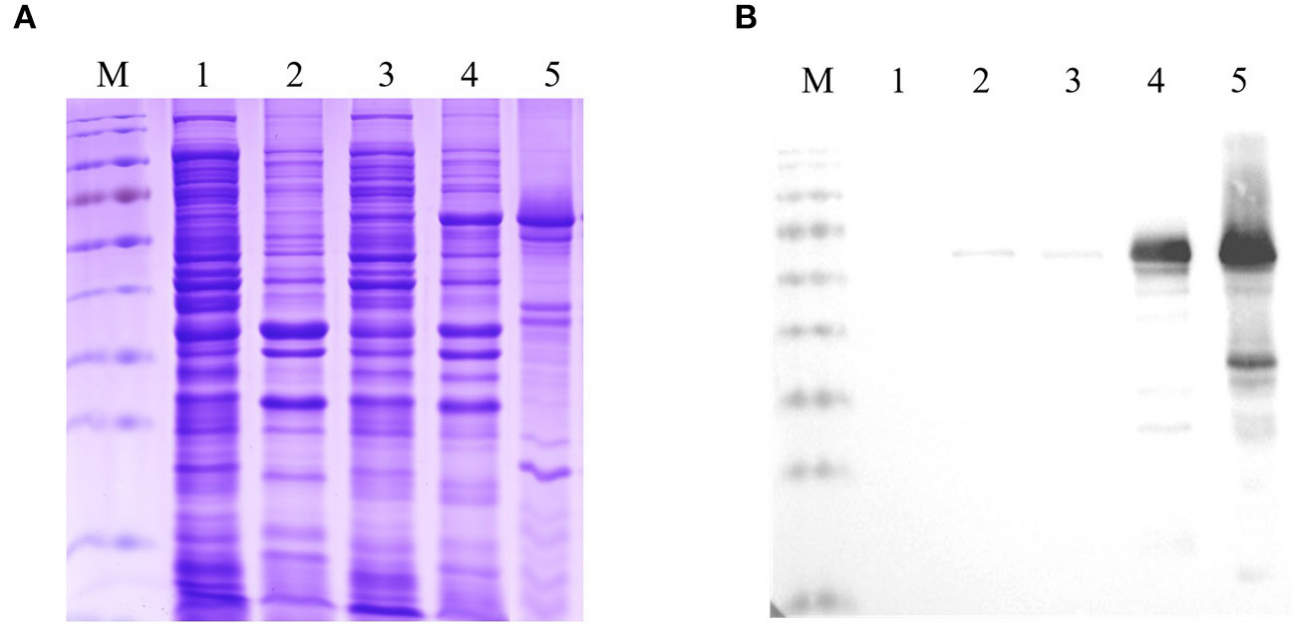
Identification of the expression and purification of the recombinant Fiber-1. **(A)** SDS-PAGE analysis for the expression and purification of the recombinant Fiber-1 in *E. coli*. **(B)** Western blot analysis for the expression and purification of the recombinant Fiber-1 by using anti-His mAb. Lane M: Protein Marker. Lane 1 and 2: the supernatant and the precipitation of the lysate of *E. coli* BL21 transfected with pCold-I, respectively. Lane 3 and 4: the supernatant and the precipitation of the lysate of *E. coli* BL21 transfected with pCold-I-Fiber-1, respectively. Lane 5: the purified Fiber-1 using urea dialysis.

To generate the recombinant Fiber-1, the recombinant plasmid pCold-I-Fiber-1 carrying the *fiber-1* gene of DAdV-3 was constructed and transformed into an *E. coli* BL21 strain. The expression of Fiber-1 in the transformed BL21 cells were then induced by IPTG, and was purified using urea dialysis. As described in Figure 1A (SDS-PAGE) and Figure 1B (Western blot), the prokaryotic recombinant Fiber-1 protein with the expected molecular mass, mainly expressed in inclusion bodies. Although several non-specific bands could be found in the purified recombinant Fiber-1 (Figure 1A), the specificity of the purified recombinant Fiber-1 was confirmed by Western blot (Figure 1B). These data showed that the recombinant Fiber-1 of DAdV-3 was successfully expressed and purified.

### Generation of six mAbs against Fiber-1 of DAdV-3

To obtain the mAbs against Fiber-1, the purified recombinant Fiber-1 was immunized to mice. After the immunization of Fiber-1, the serum of a mouse showed antibody against Fiber-1 with the highest titer of 1:6,400 by IFA (Data not shown). After the fusion and subcloning, the positive hybridoma strains stably secreting antibodies against Fiber-1 of DAdV-3 were screened out by LMH cells infected with DAdV-3 through IFA. Ultimately, six hybridomas were obtained and designated as 1D8 (Fiber-1_1D8), 1E6 (Fiber-1_1E6), 3G6 (Fiber-1_3G6), 4G1 (Fiber-1_4G1), 4G2 (Fiber-1_4G2), and 6F10 (Fiber-1_6F10), respectively. The isotyping revealed that 4G2 was identified as IgG2b subclass, and the subclass of 1D8, 1E6, 3G6, 4G1, and 6F10 was IgG1 (Table 2).

**Table 2:** Identification of the isotype of the mAbs.

	**Isotype of the mAbs**					
	**1D8**	**1E6**	**3G6**	**4G1**	**4G2**	**6F10**
Heavy chain	IgG1	IgG1	IgG1	IgG1	IgG2b	IgG1
Light chain	λ	κ	κ	λ	κ	κ

### Characteristics of mAbs against Fiber-1 of DAdV-3

**Figure 2:**
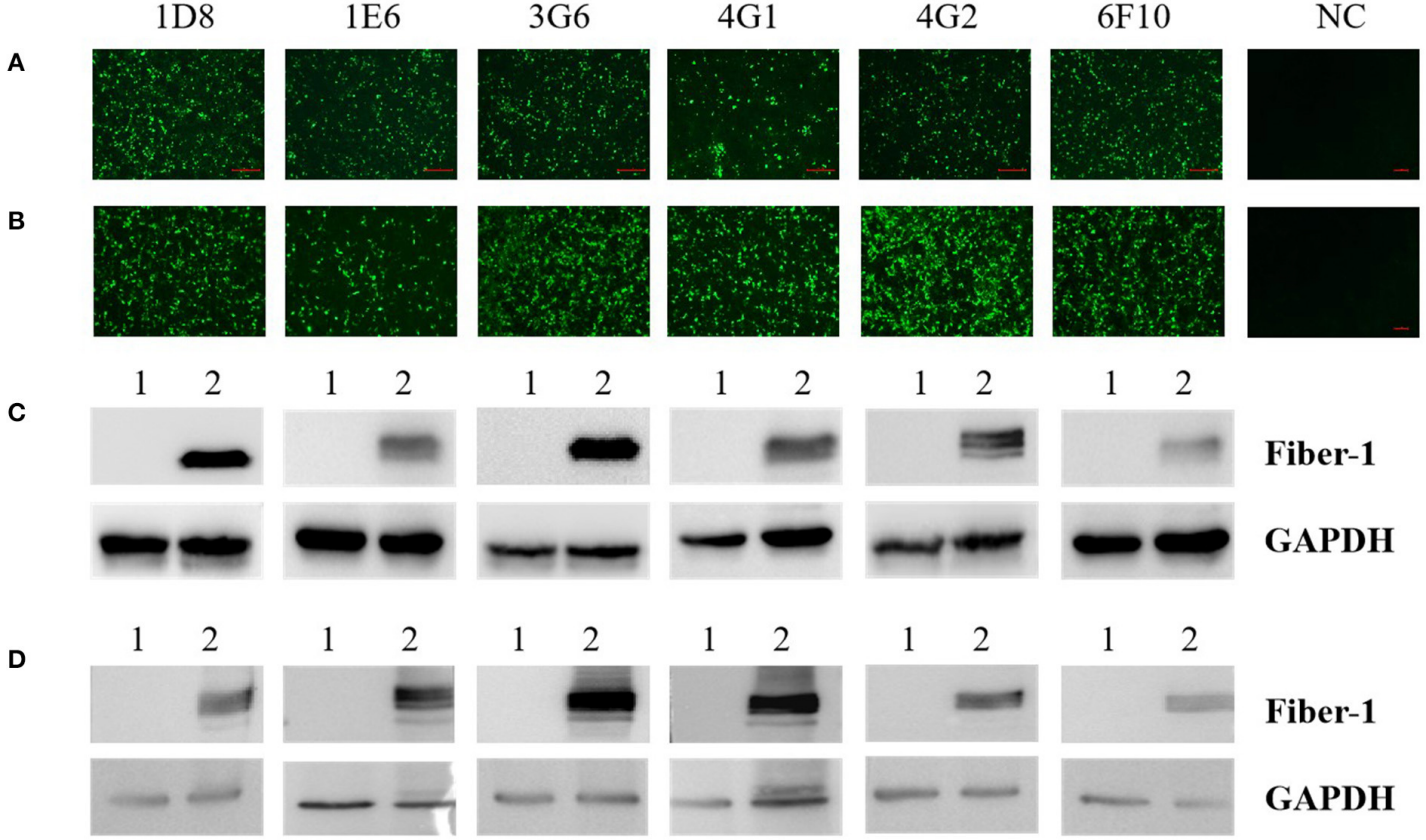
Characterization analysis of the generated mAbs by IFA and Western blot. **(A)** The mAbs reacted with LMH cells infected with DAdV-3, but not with mock-infected LMH cells (NC) by IFA. **(B)** The mAbs reacted with LMH cells transfected with pcDNA3.1-Fiber-1, but not with LMH cells transfected with pcDNA3.1 (NC) by IFA. **(C)** The mAbs reacted with LMH cells infected with DAdV-3 by Western blot. Lane 1 and 2: LMH cells infected without or with DAdV-3, respectively. **(D)** The mAbs reacted with LMH cells transfected with pcDNA3.1-Fiber-1 by Western blot. Lane 1 and 2: LMH cells transfected with pcDNA3.1 or pcDNA3.1-Fiber-1, respectively.

**Figure 3:**
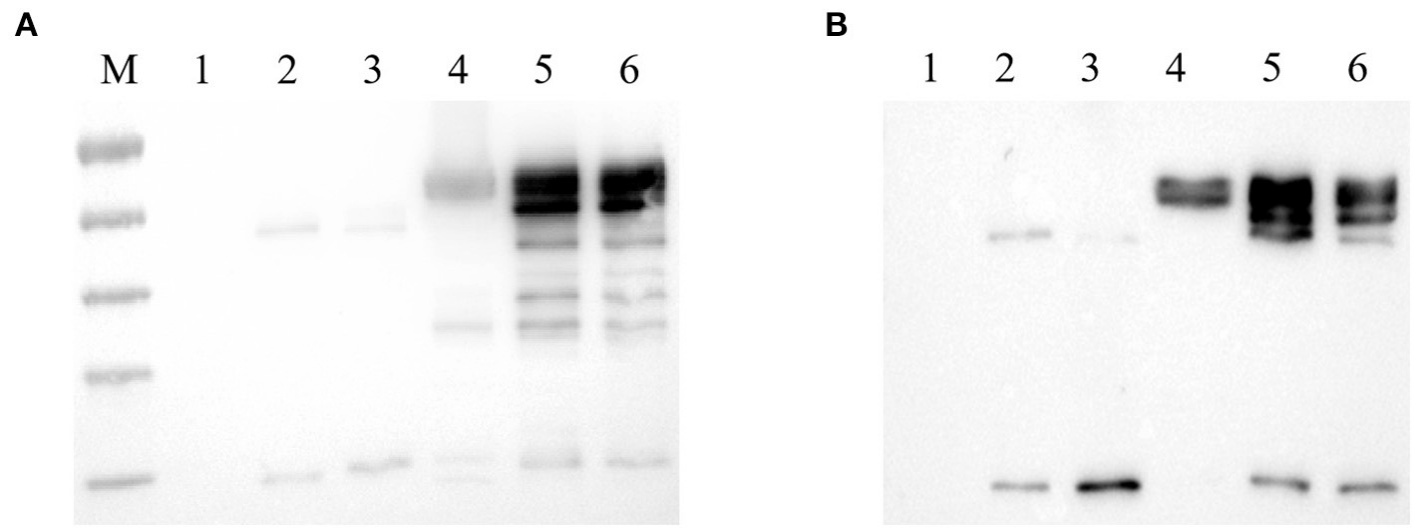
mAbs 3G6 and 6F10 efficiently immunoprecipitated Fiber-1 of DAdV-3. **(A)** mAbs 3G6 and 6F10 immunoprecipitated Fiber-1 in LMH cells transfected with pcDNA3.1-Fiber-1. Lane 1 and 4: Western blot assay for the lysates of LMH cells transfected with pcDNA3.1 and pcDNA3.1-Fiber-1, respectively. Lanes 2 and 3: Western blot assay for the lysates of LMH cells transfected with pcDNA3.1 immunoprecipitated by mAb 3G6 and 6F10, respectively. Lanes 5 and 6: Western blot assay for the lysates of LMH cells transfected with pcDNA3.1-Fiber-1 immunoprecipitated by mAb 3G6 and 6F10, respectively. **(B)** mAbs 3G6 and 6F10 immunoprecipitated Fiber-1 in LMH cells infected with DAdV-3. Lane 1 and 4: Western blot assay for the lysates of LMH cells infected without and with DAdV-3, respectively. Lanes 2 and 3: Western blot assay for the lysates of LMH cells without the infection of DAdV-3 immunoprecipitated by mAb 3G6 and 6F10, respectively. Lanes 5 and 6: Western blot assay for the lysates of LMH cells infected with DAdV-3 immunoprecipitated by mAb 3G6 and 6F10, respectively.

**Figure 4:**
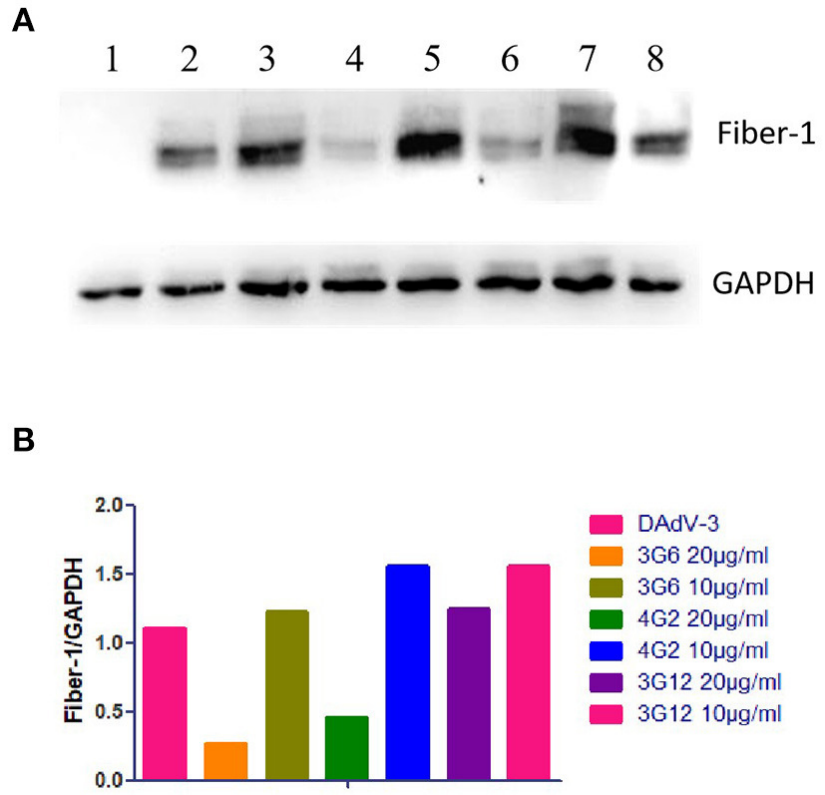
Neutralization activities of mAbs 3G6 and 4G2 against DAdV-3. **(A)** Western blot assay for the neutralizing activity of mAbs 3G6 and 4G2. Lanes 1 and 2: The lysates of LMH cells infected without or with DAdV-3. Lane 3 and 4: The lysates of the infected LMH cells treated with 10 and 20 μg/ml of mAb 3G6, respectively. Lane 5 and 6: The lysates of the infected LMH cells treated with 10 and 20 μg/ml of mAb 4G2, respectively. Lane 7 and 8: The lysates of the infected LMH cells treated with 10 and 20 μg/ml of the control mAb 3G12, respectively. **(B)** Analysis of the rate of the expressed level of Fiber-1 and GAPDH in the neutralizing test.

To evaluate the specificity of mAbs against Fiber-1 generated, the LMH cells infected with DAdV-3 or transfected with pcDNA3.1-Fiber-1 were tested by these mAbs through IFA and Western blot. As described in Figure 2, the six mAbs could specifically recognize the expressed Fiber-1 protein in LMH cells infected with DAdV-3 or transfected with pcDNA3.1-Fiber-1, but not react with mock-infected LMH cells or pcDNA3.1-transfected LMH cells. The specific green fluorescence in IFA could be found and the specific band for Fiber-1 in Western blot could be recognized by these mAbs in LMH cells infected with DAdV-3 or transfected with pcDNA3.1-Fiber-1, but not in LMH cells or LMH cells transfected with pcDNA3.1. Moreover, the purified mAbs 3G6 and 6F10, but not mAbs 1D8, 1E6, 4G1, and 4G2, could efficiently immunoprecipitate the Fiber-1 protein in LMH cells either infected with DAdV-3 or transfected with pcDNA3.1-Fiber-1 as described in Figure 3, highlighting that the mAbs 3G6 and 6F10 would provide powerful tools for identifying the interaction between Fiber-1 and host proteins. Notably, among these mAbs, 3G6 and 4G2 showed certain neutralizing activity for inhibiting/blocking the infection or replication of DAdV-3 in LMH cells. As described in Figure 4, the protein rate of Fiber-1 and GAPDH in LMH cells with the treatment of 20 μg/ml of 3G6 and 4G2 was decreased by about 4- and 2-folds, respectively, in comparison with that in LMH cells with the treatment of 20 μg/ml of the control mAb 3G12 or LMH cells only infected with DAdV-3.

### Identification of novel B cell epitopes in Fiber-1 of DAdV-3

**Figure 5:**
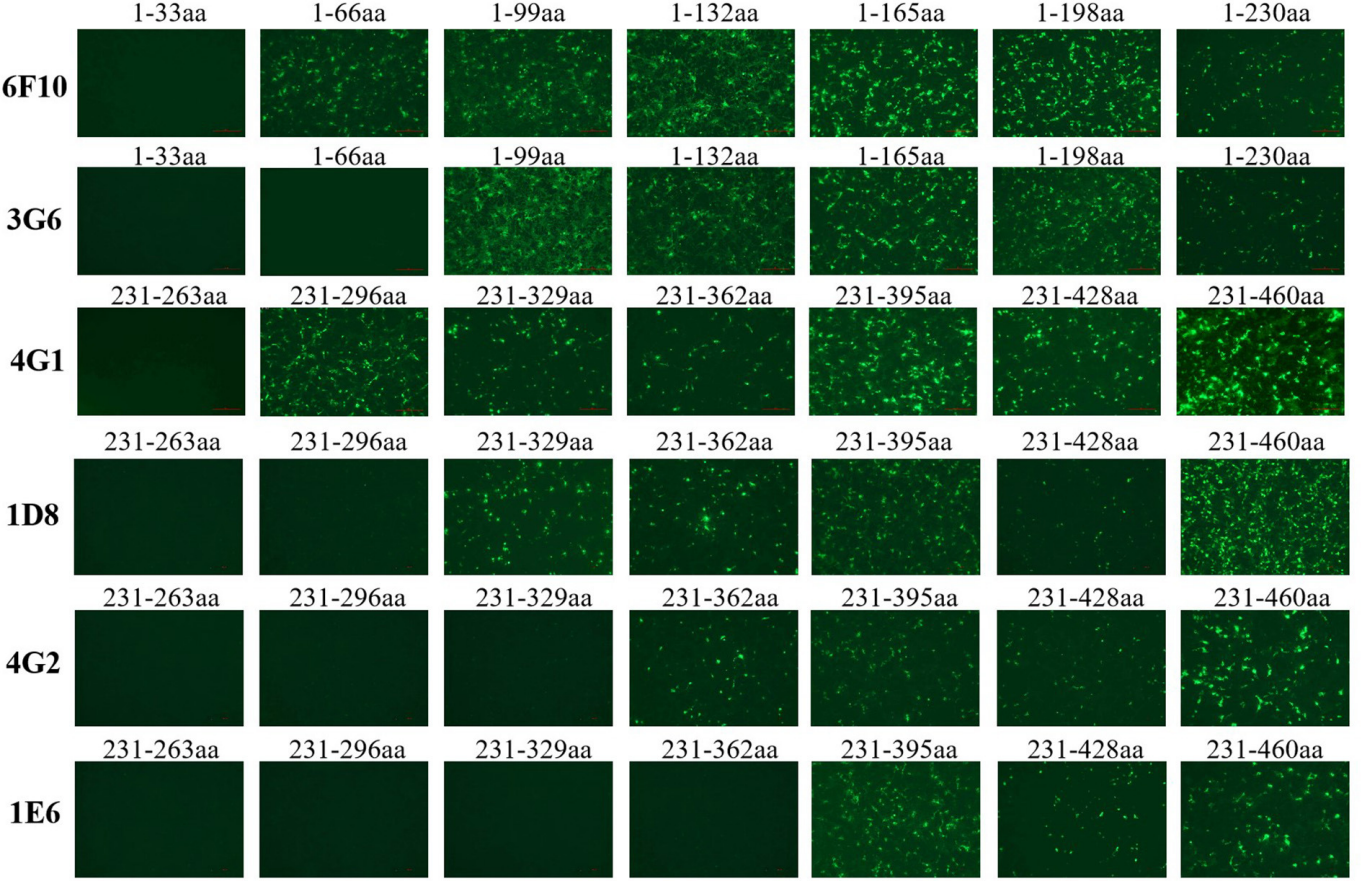
Mapping the epitopes in Fiber-1 protein recognized by these mAbs. LMH cells transfected with the different Fiber-1 truncations were tested using mAb 6F10, 3G6, 4G1, 1D8, 4G2, and 1E6 by IFA.

**Figure 6:**
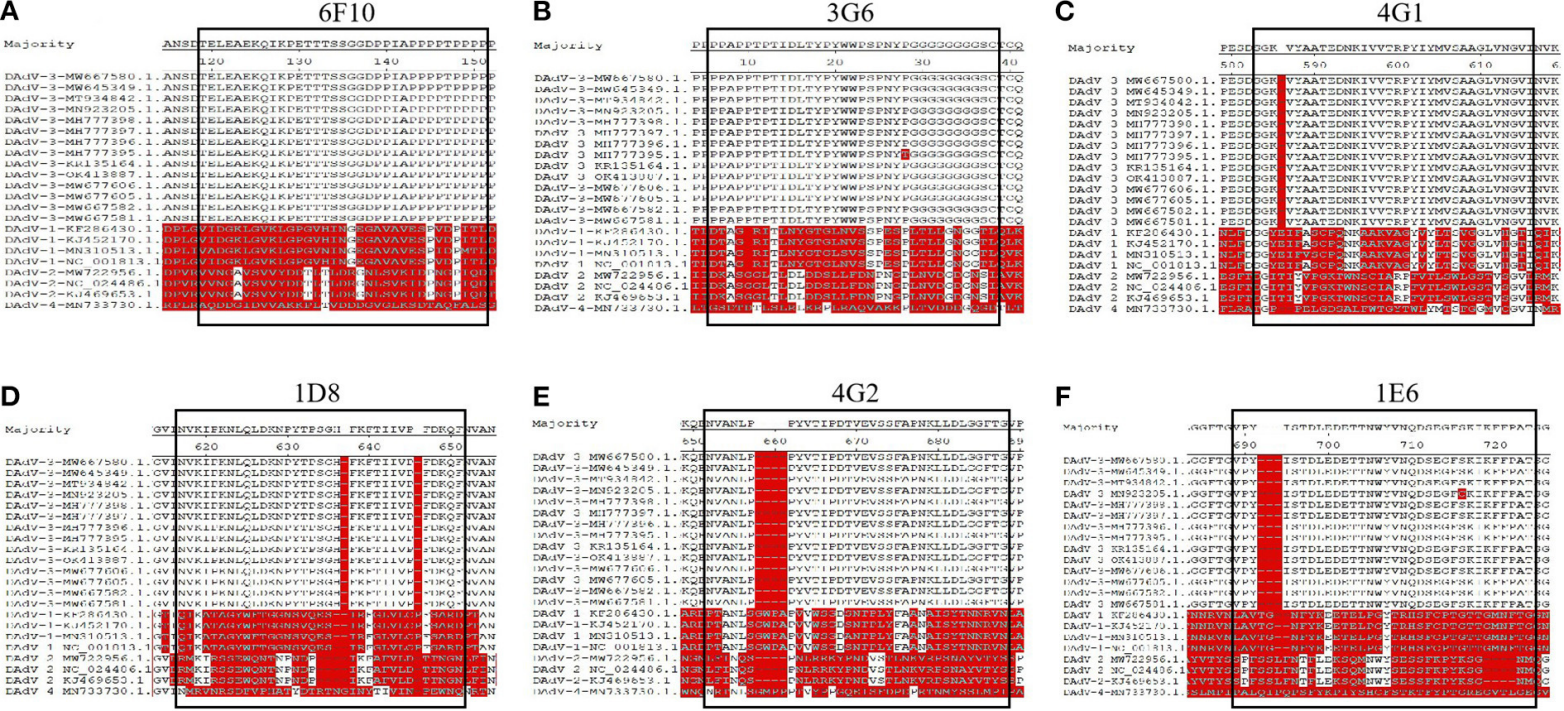
Sequence alignment analysis for the identified epitopes in Fiber-1. The amino acid sequence analysis of the epitopes recognized by 6F10 **(A)**, 3G6 **(B)**, 4G1 **(C)**, 1D8 **(D)**, 4G2 **(E)**, 1E6 **(F)** with different DAdV-3 and other representive DAdV isolates.

To identify the epitopes recognized by these mAbs generated here, a serial of truncations of *fiber-1* gene were constructed according to the primers listed in Table 1. The recombinant plasmids with different *fiber-1* gene truncations were transfected into LMH cells and the B cell epitopes recognized by mAbs were mapped by IFA. As described in Figure 5, the epitope recognized by mAbs 6F10, 3G6, 4G1, 1D8, 4G2, and 1E6 was located in 34-66aa, 67-99aa, 264-296aa, 297-329aa, 330-362aa, and 363-395aa of Fiber-1, respectively. To analyze the conservation of the identified epitopes, the sequences of Fiber-1 among different DAdV-3 and other representive DAdV isolates were aligned. As shown in Figure 6, the B cell epitopes identified here were highly conserved among different DAdV-3 isolates, but not in other DAdV isolates, highlighting the specificity of these mAbs against the Fiber-1 of DAdV-3.

## Discussion

DAdV-3, characterized by swelling and hemorrhagic liver and kidney in Muscovy duck flocks, broke out in China in 2014. To date, multiple similar cases have been reported in Guangdong, Fujian, Zhejiang, Anhui, Jiangsu provinces (5, 7). Recently, DAdV-3 mutants with the truncated ORF67 were reported, spread and became prevalent (8). Such novel DAdV-3 isolates may be more pathogenic, suggesting that more attention should be paid on monitoring the infection and epidemiology of DAdV-3 (9). In previous studies, Fiber-2 of FAdV-4 as a vital surface protein was related to viral virulence, and Fiber-1 of FAdV-4 was proven to directly mediate the virus infection (10–12). However, the roles of the Fiber-1 and Fiber-2 of DAdV-3 in the infection and pathogenesis of DAdV-3 need to be elucidated.

In this study, the recombinant Fiber-1 of DAdV-3 was first expressed in *E. coli* and purified. And then, six mAbs, designated as 1D8, 1E6, 3G6, 4G1, 4G2, and 6F10, were generated using the purified Fiber-1 as an immunogen. Notably, all these mAbs could react with the expressed Fiber-1 in LMH cell either infected with DAdV-3 or transfected with pcDNA3.1-Fiber-1 through both IFA and Western blot, also highlighting the efficient antigenicity of the Fiber-1 protein expressed in *E. coli*. Notably, we did immunoprecipitation using all these six mAbs (data not shown), but we only found that mAb 3G6 and 6F10 could immunoprecipitate Fiber-1 (Figure 3). This might be related to the different epitopes recognized by these different mAbs or the different affinity between Fiber-1 and mAbs. mAbs 3G6 and 6F10 could immunoprecipitate the Fiber-1 in LMH cells either infected with DAdV-3 or transfected with pcDNA3.1-Fiber-1, suggesting mAbs 3G6 and 6F10 could be used for the identification of host proteins interacted with Fiber-1 of DAdV-3. The Western blot data indicated that all these epitopes recognized by these mAbs were linear. Epitope mapping demonstrated that mAbs 1D8, 1E6, 3G6, 4G1, 4G2, and 6F10 recognized different epitopes in the Fiber-1 including 34-66aa, 67-99aa, 264-296aa, 297-329aa, 330-362aa, and 363-395aa. Sequence analysis further revealed that B cell epitopes identified in the study were highly conserved in DAdV-3, but not in other serotypes of DAdV, indicating these mAbs had potential to be applied to the development of specific diagnostic methods for detection of DAdV-3. In the neutralizing assay, although mAbs 3G6 and 4G2 showed certain blocking activity against the infection of DAdV-3 in LMH cells (other four mAbs had no blocking activity against DAdV-3, data not shown), the neutralizing activity of mAbs 3G6 and 4G2 was not high, indicating that the Fiber-1 plays vital roles in mediating the infection of DAdV-3 whereas the conformation epitopes, but not the linear epitopes identified here, may play significant roles in inducing neutralizing antibodies against DAdV-3. It is also possible that mAbs 3G6 and 4G2 could recognize the conformational protein of Fiber-1 with the exposure linear epitopes.

To our knowledge, this is the first demonstration of the generation of six mAbs specific to the Fiber-1 of DAdV-3 and the identification of six novel B cell epitopes in the Fiber-1. The generated mAbs against the Fiber-1 and their defined B cell epitopes in the Fiber-1 would provide valuable tools for further studying on the interacted host proteins of Fiber-1 and the development of serological diagnostic approaches specific for DAdV-3. The ELISA approaches for detection of antibody or antigen of DAdV-3 using these mAbs are underdeveloped.

## Data availability statement

The original contributions presented in the study are included in the article/supplementary material, further inquiries can be directed to the corresponding authors.

## Ethics statement

All animal experiments were performed in accordance with the Institutional Animal Care Guidelines and the Protocol (No. SYXY-3) which was approved by the Animal Care Committee of Yangzhou University in China.

## Author contributions

JY, HS, and AQ designed the project. WZ, HS, YL, and JX carried out the experiments. HS, WZ, DR, JY, and ZW analyzed the data. HS, WZ, QX, and TL drafted the manuscript. JY and AQ discussed and prepared the final report. All authors have read and approved the final manuscript.

## Funding

This study was funded by Postgraduate Research and Practice Innovation Program of Jiangsu Province (SJCX22_1807), Key Laboratory of Prevention and Control of Biological Hazard Factors (Animal Origin) for Agrifood Safety and Quality (26116120), Research Foundation for Talented Scholars in Yangzhou University, and the Priority Academic Program Development of Jiangsu Higher Education Institutions.

## Conflict of interest

The authors declare that the research was conducted in the absence of any commercial or financial relationships that could be construed as a potential conflict of interest.

## Publisher's note

All claims expressed in this article are solely those of the authors and do not necessarily represent those of their affiliated organizations, or those of the publisher, the editors and the reviewers. Any product that may be evaluated in this article, or claim that may be made by its manufacturer, is not guaranteed or endorsed by the publisher.
